# Pterygoid Hamulus: Morphological Analysis and Clinical Implications

**DOI:** 10.7759/cureus.55694

**Published:** 2024-03-06

**Authors:** Nymfodora Malkidou, Konstantinos Chaidas, Vasilios Thomaidis, Katerina Vassiou, Aliki Fiska

**Affiliations:** 1 Anatomy, Democritus University of Thrace, Alexandroupolis, GRC; 2 Otolaryngology, University Hospital of Alexandroupolis, Democritus University of Thrace, Alexandroupolis, GRC; 3 Anatomy, Faculty of Medicine, University of Thessaly, Larissa, GRC

**Keywords:** pterygoid hamular syndrome, pterygoid hamular measurements, pterygoid hamulus fracture, obstructive sleep apnea, middle ear ventilation

## Abstract

Introduction:* *The pterygoid hamulus (PH), as a small and curved projection of the sphenoid bone, occupies a unique position at the skull base. Given its functional relation with the surrounding anatomical structures, the study of this rather underrepresented structure in the literature assumes paramount importance.

Materials and methods: We examined a total of 87 pterygoid hamuli (50 right-sided and 37 left-sided) out of a sample of 114 dry skulls. We measured the length, width, and angle of each PH and the interpterygoid distance in skulls with both pterygoid hamuli intact, and we calculated the mean, maximum, and minimum values.

Results: Our statistical analysis revealed the mean length (0.9 cm), width (0.3 cm), and angle (47.8°) of the PH, as well as the mean interpterygoid distance (3.31 cm). We recorded the longest-ever documented PH (1.64 cm). The obtained length values were higher than those provided by radiological studies. We also investigated possible associations between anatomy and pathological conditions related to the PH morphology, including pterygoid hamular elongation syndrome, hamular fracture, middle ear disorders, and obstructive sleep apnea syndrome.

Conclusion: Our study uses precise measurement techniques to detail the anatomy of the PH in dry skulls. This research can be a valuable resource for future studies, advancing our understanding of the PH's structure and its clinical significance.

## Introduction

The pterygoid hamulus (PH) is a curved, small, and slender bony protrusion that arises from the base of the medial pterygoid plate of the sphenoid bone, extending downward and then turning laterally. Although its parts, i.e., base, body, neck, and head, are described in anatomical texts, they cannot be distinguished with the naked eye [1,2]. Situated at a pivotal point, the PH supports several structures, including the tensor veli palatini muscle, the pterygomandibular ligament, the upper part of the pterygopharyngeal sphincter muscle (a component of the upper pharyngeal constrictor muscle), and the buccinator muscle. The tendon of the tensor veli palatini muscle wraps around a sharp bend in the neck of the PH to alter its course and insert into the palatine aponeurosis. A small bursa between the muscle tendon and the groove on the neck of the PH ensures smooth movement of the soft palate. Contraction of the muscle around the hamulus separates the oral and nasal cavities and equalizes air pressure in the middle ear during activities such as sucking, swallowing, chewing, sneezing, and yawning. The upper pharyngeal sphincter contributes to these functions and also plays a role in maintaining airway patency during sleep [3-5]. Despite its significant functions, the PH remains relatively underrated on the anatomical chart due to its small size and varying morphology. We aim to thoroughly study its structure in dry skulls from a purely anatomical point of view and consider its clinical implications.

## Materials and methods

We studied 114 adult human dry skulls of unknown sex and of Caucasian (Hellenic) origin, collected from the Anatomy Laboratory of Democritus University of Thrace in Alexandroupolis, Greece. Only 57 skulls, constituting half of the sample, had at least one intact PH and were included in the measurements. In total, we measured 87 pterygoid hamuli, i.e., 50 on the right and 37 on the left side. Thirty of them had intact pterygoid hamuli bilaterally. Two hamuli with evident fracture healing were excluded from further evaluation. Two researchers independently conducted all measurements using a digital vernier caliper with a precision of 0.01 cm. For each difference measured, the average of two values was calculated.

**Figure 1:**
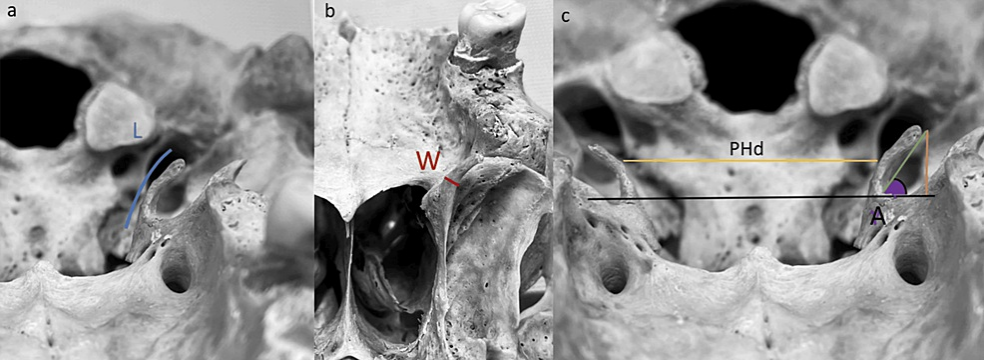
Measurements of the PH in dry skulls a: Length (L) from the base to the head of the PH (blue line) b: Width (W), i.e., the maximum transverse distance (red line) c: Angle (A) is indicated by the sine of the acute angle of PH (purple) + orange line indicating the vertical distance between the hamulus head and the line joining the two bases of the hamuli + green line, which is the direct line between the head and the base of the PH; the yellow line is the interpterygoid distance (PHd) between the right and left PH PH: Pterygoid hamulus

The morphological analysis (Figure 1) included four parameters. Length is the distance from the base to the head of the PH, measured at its outer perimeter. Width refers to the maximum transverse distance. The angle involves the sine of the acute angle of the PH. The numerator was the vertical distance between the hamulus head and the line joining the two bases of the hamuli, which corresponds to the horizontal plane, and the denominator was the direct line between the head and the base of the PH, i.e., the curve of its curvature. The interpterygoid distance indicates the distance between the medial sides of the right and left PH (at the level of the maximum transverse distance of each PH).

Data were presented as mean ± standard deviation (SD), including values below (<mean-SD) and above (>mean+SD), or as a percentage. The Wilcoxon signed-rank test was used to compare the anatomical parameters between the left and right PH of the skulls, with both pterygoid hamuli intact. The Pearson correlation test was employed to assess potential correlations between the length and width of the PH, as well as potential correlations between the interpterygoid distance and the length or width of the PH. A p-value < 0.05 was considered statistically significant. All data were statistically analyzed using SPSS Statistics version 27.0 (IBM Corp., Armonk, NY, USA).

## Results

The length of the pterygoid hamuli ranged between 0.5 cm and 1.64 cm. The maximum length was observed on the left side, while the minimum was observed bilaterally. Length values higher than the mean + SD were reported in almost twice the number of hamuli on the left side (16%) compared to the right side (9%). The right-sided pterygoid hamuli recorded both the highest (0.5 cm) and the lowest width (0.12 cm). One-fourth of the left-sided hamuli (26%) presented angle values higher than the mean + SD; the maximum angle (74.50) was included among them. The minimum angle (15.30) was observed on a right-sided PH, but at the same time, half of them (43%) had angle values greater than the mean + SD (Table 1).

**Table 1:** Measurements of the length, width, angle, and interpterygoid distance of the pterygoid hamuli SD: Standard deviation, PH: Pterygoid hamulus

Parameters	Mean ± SD	Μinimum		Maximum	
		Right	Left	Right	Left
Length of PH (cm)	0.90 ± 0.22	0.50	0.50	1.25	1.64
Width of PH (cm)	0.30 ± 0.09	0.12	0.14	0.50	0.48
Angle of PH (^0^)	47.8 ± 8.8	15.30	18.10	70.60	74.50
Interpterygoid distance (cm)	3.31 ± 0.33	2.71		4.01	

**Figure 2:**
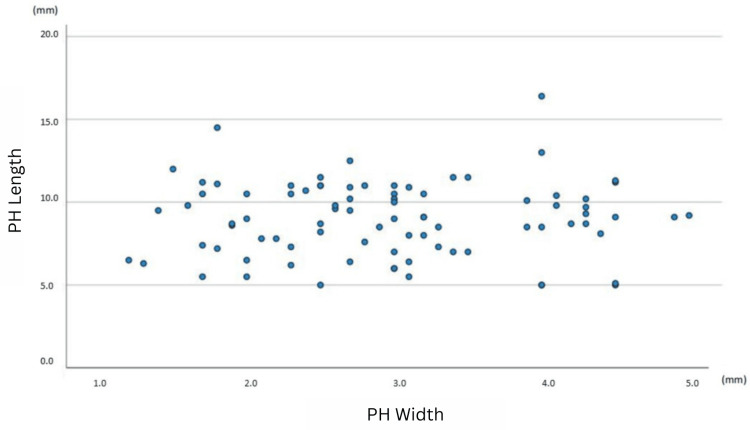
A scatterplot of PH length vs. PH width for the total sample of 87 pterygoid hamuli Each dot represents a single PH. Each point’s horizontal position indicates the PH length (in mm) and the vertical position indicates the PH width (in mm). PH: Pterygoid hamulus

**Figure 3:**
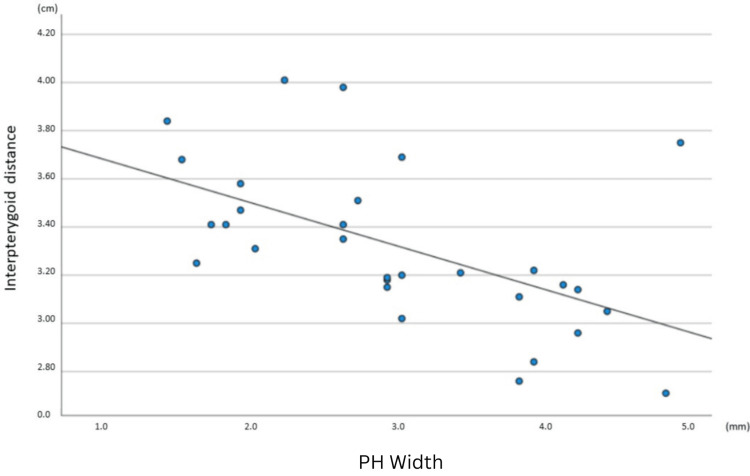
A scatterplot of interpterygoid distance vs. PH width for the sample of 30 skulls with intact bilateral PH Each dot represents the anatomic measurements of a single skull. Each point’s horizontal position indicates the interpterygoid distance (in cm) and the vertical position indicates the PH width (in mm). The line of best fit indicates the presence of a negative correlation (r = -0.555, p = 0.001). PH: Pterygoid hamulus

A comparison using Pearson’s correlation revealed that there is no statistically significant association between either the length and width of the pterygoid hamuli (r = 0.045, p = 0.681) (Figure 2) or the length of the PH and the interpterygoid distance (r = 0.201, p = 0.288). In contrast, a statistically significant negative correlation between the width of the PH and the interpterygoid distance was identified (r = -0.555, p = 0.001) (Figure 3). Furthermore, there was a statistically significant positive correlation between the angle of the PH and the interpterygoid distance (r = 0.404, p = 0.027). Comparison of the length, width, and angle between the left and right sides of the 30 skulls with both pterygoid hamuli intact yielded no statistically significant differences (Table 2).

**Table 2:** Comparison of length, width, and angle of pterygoid hamuli on skulls with intact bilateral PH (n=30) PH: Pterygoid hamulus

Parameters	Μean ± SD (minimum to maximum)		p-value
	Right-sided PH	Left-sided PH	
Length (cm)	0.9 ± 0.2 (0.5 - 1.25)	0.9 ± 0.2 (0.5 - 1.64)	0.336
Width (cm)	0.3 ± 0.1 (0.15 - 0.5)	0.3 ± 0.1 (0.14 - 0.48)	0.983
Angle (^0^)	49.2 ± 13.8 (15.3 - 70.6)	47.1 ± 12.9 (18.1 - 74.5)	0.270

## Discussion

Comparison of measurements in pterygoid hamulus

Prior morphological investigations of the PH focused on its length, width, and angle, employing CT and cone-beam CT (CBCT) [2,6-11]. The anatomical study by Putz and Kroyer served as the foundational reference for subsequent anatomical and radiological inquiries into the PH [1]. Our comprehensive anatomical analysis differs from previous works (Table 3) in terms of methodological detail and accuracy. We specify the number of dry skulls and pterygoid hamuli examined, as well as the exact measurement method. Additionally, we provide information about the maximum and minimum values for length, width, and angle. Seemingly small differences, measuring only a few millimeters, carry major significance for such a minute anatomical structure as the PH. In comparison with the anatomical studies of Putz and Kroyer [1] and Krmpotić-Nemanić et al. [4], we obtained a slightly higher mean length value (0.9 cm vs. 0.7 cm). On the other hand, radiological studies documented lower mean lengths, ranging from 0.45 cm to 0.69 cm [2,6-9,11].

**Table 3:** Comparative analysis of measurement methods and outcomes for the PH in the literature PH: Pterygoid hamulus, CBCT: Cone-beam computed tomography, R: Right, L: Left, PHd: Interpterygoid distance

Authors	No. of pterygoid hamuli	Method	Mean length (cm)	Maximum length (cm)	Minimum length (cm)	Mean Width (cm)	Maximum width (cm)	Minimum width (cm)	Mean angle (^o^)	Mean PHd (cm)
Eyrich et al. [6]	40	CT	R:0.49, L:0.5	0.8	0.3	-	-	-	-	3.89
Putz et al. [1]	-	Dry skulls	0.72	-	-	0.14	-	-	58	3.06
Krmpotić-Nemanić et al. [4]	-	Dry skulls	6.97± 1.7	-	-	-	-	-	-	2.73 to 4.07
Orhan et al. [8]	396	CBCT	R:0.54 ± 0.2, L:0.54 ±0.19	1.09	-	R:0.18 ±0.11, L:0.17±0.09	-		-	-
Oz et al. [9]	100	CBCT	R:0.45 ±0.20, L:0.41 ±0.16	-	-	R:0.17 ±0.05, L:0.16 ±0.04	-	-	-	-
Komarnitki et al. [10]	-	CBCT	0.67 ± 0.21	-	-	0.22 ± 0.05	-	-	-	-
Komarnitki et al. [2]	100	CBCT	0.688 ± 0.22	1.2	0.09	0.18 ± 0.55	0.38	0.08	-	-
Kuzucu et al. [7]	174	CT	R:0.56 to 0.62, L:0.56 to 0.64	-	-	R:0.15 to 0.18, L:0.15 to 0.18	-	-	-	2.75 to 3.26
Mehra et al. [11]	2000	CBCT	R:0.67 ±0.17, L:0.69 ±0.16	-	-	R:0.16 ±0.04, L:0.17 ±0.03	-	-	-	-
Piccin et al. [12]	-	CT	-	-	-	-	-	-	-	2.93 to 3.42
Our study	87	Dry skulls	0.90 ± 0.22	1.64	0.5	0.302 ± 0.099	0.5	0.12	47.8 ± 8.8	3.31 ± 0.33

**Figure 4:**
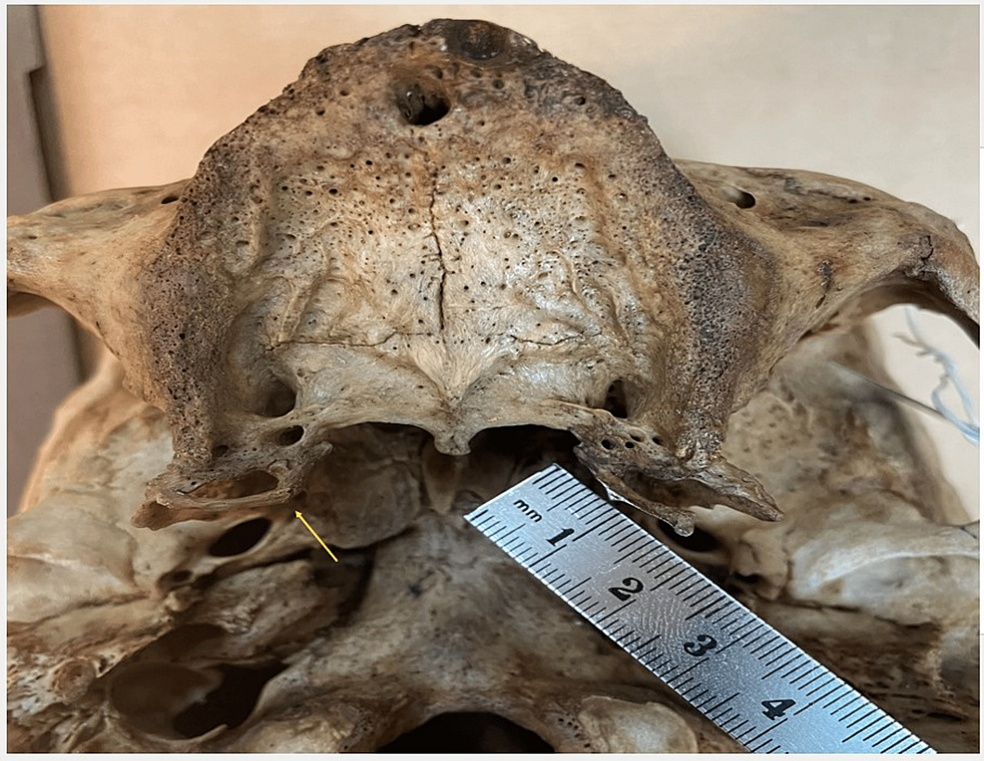
Length of a PH measuring 1.64 cm Seen here is a fracture of the contralateral PH with obvious bony callus formation (yellow arrow). PH: Pterygoid hamulus

Disparities in maximum length values were also evident when comparing our findings to those of previous studies [2,6]. Our study reveals a maximum length of 1.64 cm in a left-sided PH (Figure 4), representing the highest value ever documented in the literature, to our knowledge. The contralateral hamulus of the same skull is worth mentioning since it presented a healed fracture at its base and measured 1.9 cm. Due to pathology, it was not included in the present study’s sample, but it raised our concern about an unknown PH disorder: the broken PH (Figure 4) [13]. The angle measurements closely resemble those reported by Putz and Kroyer [1] and Krmpotić-Nemanić et al. [4], but the width differs (0.3 cm vs. 0.14 cm), even though we used the same tool and roughly the same level of the PH to measure it.

Imaging studies report narrower widths, ranging from 0.12 cm to 0.22 cm [2,7,8,11], following the pattern of extracting smaller length values from radiology. Furthermore, imaging studies reporting exceptionally low values for length and width (0.09 cm and 0.08 cm, respectively) [2] should raise questions about the PH stability, functionality, and ability to serve as a fulcrum for the tensor veli palatini muscle. On the contrary, the minimum length of 0.12 cm in our study is more consistent with a PH that remains functional and capable of representing a stable trochlea for the muscle and an attachment point for the upper pharyngeal sphincter. Comparing the interpterygoid distance between our study and other anatomical or radiological studies revealed similar measurement results, indicating the accuracy of imaging measurements in non-minimized structures [1,4,6,7,13].

The pterygoid hamulus in dentistry

Diagnosis and management of diseases in the hamular region are common parts of a dentist's practice. Patients complain about localized palate pain, especially when pressing the tongue against this area. They also report discomfort in the palate and the jaw, ear and throat pain, and difficulty swallowing. Intraoral examination reveals signs of edema and erythema in the anterolateral soft palatal area. Dental professionals may misdiagnose pterygoid hamular pathology as temporomandibular disorders, impacted teeth, trigeminal or glossopharyngeal neuralgia, calcification of the stylohyoid ligament, or even otitis media [14-16].

Pterygoid hamulus syndrome

The most common pathology associated with the PH, elongation syndrome, occurs in approximately 1% of cases [17]. Patients commonly exhibit a range of symptoms, including headaches, ear pain, hearing disorders, dysphagia, edema, erythema, and tenderness upon palpation of the soft palate [18-20]. In the case report by Sasaki et al., a patient diagnosed with elongated PH syndrome underwent surgical resection of the left PH. The resected PH length was 1.3 cm. The authors suggested a correlation between this length value and the manifestation of symptoms of the elongated PH syndrome [18]. However, we found three of the PH (out of the 87 examined) having lengths equal to or higher than 1.3 cm (1.64 cm, 1.45 cm, and 1.3 cm, respectively), implying an estimated 3.5% occurrence of the elongation PH syndrome. According to another study, a PH measuring more than 0.8 cm in length is considered elongated [20]. If this criterion is applied to our study, 56 out of 87 pterygoid hamuli (64.3%) would be classified as elongated; this is an unreasonably high percentage. Although the evidence is insufficient, these findings suggest that the diagnosis of the PH syndrome is multifactorial and does not depend entirely on its length. The combination of the individual's medical history, symptomatology, and the morphology of the PH will determine the presence of the syndrome.

The case reports of left-sided elongation syndrome, with a normal right-side PH [18,19], are consistent with our findings of higher length values in the left-sided pterygoid hamuli in comparison to their counterparts on the right side. Notably, the case reports of right-sided elongation syndrome do not include length measurements of the PH. Instead, they establish the diagnosis based on the presence of pain in the right hamular area [6,14,16]. Data about high right-sided PH length (exceeding 1.3 cm) are absent, including in our study, where the highest measured value was 1.25 cm.

Another aspect of the PH syndrome involves the inflammation of the bursa known as pterygoid hamular bursitis, which impacts the adjacent soft tissues [21,22]. This bursa is situated within the groove of the PH’s head and plays a vital role in facilitating the movement of the tensor veli palatini muscle around the hamulus during contraction [15]. The examination of all the pterygoid hamuli in our dry skulls did not reveal any pathological or degenerative changes associated with pterygoid hamular bursitis, such as osteophytes.

The PH syndrome is prone to misdiagnosis, often being confused with various other conditions due to the overlapping anatomical structures within a confined area. Eagle's syndrome, temporomandibular disorders (TMDs), trigeminal ganglion neuralgia, glossopharyngeal neuralgia, cysts and tumors of the area, otitis media, foreign bodies, burning mouth syndrome, and impacted third molars fall into the differential diagnosis. Conversely, sporadic or atypical facial pain in the hamular area may be attributed to PH syndrome, whereas it is most usually due to the traction of the tensor veli palatini muscle or minimal trauma to the adjacent soft tissues. These diagnostic challenges can significantly hinder therapeutic interventions, potentially leading to the chronicity of the syndrome [8,15].

Pterygoid hamulus fracture

Kane et al. and Schönmeyr et al. discussed PH fracture as a medical procedure performed during palatoplasty to release tension of the tensor veli palatini muscle in cleft palate patients [23, 24]. However, we found no reports of the PH fracture as a clinical entity, neither in anatomical nor radiological studies. Interestingly, in the dry skulls we examined, we observed PH fractures in two out of the 87 PH, raising its incidence to 2.3% (Figure 4). We assumed that the most probable causative mechanism of the fractures may be the extraction of the third maxillary molar tooth, as it requires forceful surgical manipulations in its proximity [13]. Orhan et al. reported such a case, in which symptoms of PH pathology manifested following a third molar tooth extraction [8]. Any fracture of the PH would lead to functional instability in the adjacent muscle and tendon attachments. However, if it doesn’t lead to an avulsion fracture, bone healing would restore the anatomy and function of the PH. This may explain the absence of PH fracture reports in the medical literature.

Abnormal middle ear ventilation

The tensor veli palatini and tensor tympani act simultaneously and synergistically to increase intratympanic pressure temporarily. Specifically, the dilator tubae, a subunit of the tensor veli palatini, is responsible for dilating the eustachian tube [25]. Dysfunction of the tensor veli palatini can disrupt the intratympanic environment, leading to increased pressure and resulting in vertigo, tinnitus, otalgia, hypoacusis, and a sensation of fullness [22]. Schönmeyr et al. and Barchetta et al. reported eustachian tube dysfunction and a high incidence of otitis media in cases of cleft palate, while Kane et al., studying the outcomes of an iatrogenic PH fracture after palatoplasty, concluded that the middle ear environment remains unaffected [15,23,24]. Ramirez et al. reported that pterygoid hamular bursitis can produce symptoms in the middle ear [21].

Okada et al. found that the tensor veli palatini muscle fibers divide into two layers in their inferior portion: the medial layer turns into the PH, and the lateral layer distributes to the hamulus and connects to the buccinator muscle. However, they did not provide information about the dilator tubae part of the muscle [26]. According to this study, a fracture of the hamulus or bursitis would not affect the middle ear due to the multiple directions of the fibers of the tensor veli palatini. To establish a clear connection between PH pathology and middle ear manifestations, one should investigate only the dilator tubae fibers that act on the tube.

Obstructive sleep apnea syndrome

Obstructive sleep apnea syndrome (OSAS) is characterized by recurrent episodes of partial or complete upper airway collapse during sleep, leading to intermittent airflow limitation, sleep fragmentation, arterial oxygen desaturations, and poor sleep quality. Multi-level upper airway collapse is often present, and several factors, including airway anatomy and reduced pharyngeal muscle activity, can play a role [3].

The PH has a significant impact on normal upper airway function; it influences the activity of the soft palate muscles and prevents pharyngeal collapse at the velopharyngeal level, acting as a pivotal point for several muscles’ activity. Krmpotić-Nemanić et al. and Orhan et al. suggested that the PH atrophies with age, and its small size could potentially contribute to the narrowing of the upper pharynx as it fails to provide adequate muscular support. This structural insufficiency can lead to disordered breathing during sleep and obstructive sleep apnea [4,8]. According to our data, 10 out of the 87 pterygoid hamuli were of small size (length and width values lower than the mean - SD); this suggests that approximately 11.5% of individuals may experience sleep-related breathing problems attributed to the PH size. Oz et al. associated the severity of obstructive sleep apnea syndrome with reduced PH length [9]. Adhering to these criteria, our study's measurements would set the estimated occurrence of OSAS at 17% (15 out of 87 pterygoid hamuli). On the other hand, Kuzucu et al. suggested that the length of the PH is not a strong factor for predicting patients with OSAS. Instead, the authors linked the severity of OSAS with the increasing thickness of the PH and the decreasing interpterygoid distance [7]. Piccin et al. confirmed that a narrow interpterygoid distance constitutes a contributing factor to the development of OSAS [13].

Our study revealed the presence of a statistically significant negative correlation between the interpterygoid distance and the PH width, both considered predisposing factors for OSAS, at an incidence rate of 13% (four out of 30 skulls). A statistically significant negative correlation also existed between the interpterygoid distance and the PH angle, which is reasonable, whereas there was no significant association between the interpterygoid distance and the PH length. Considering these findings, further studies to evaluate the anatomical characteristics of the PH and their role in the complex pathophysiology of OSA will be required to clarify the association between OSAS and PH anatomy and potentially guide targeted treatment.

Strengths and limitations of the study

Dry skulls offer a standardized and consistent medium for measurements, reducing variability and errors that may arise from factors inherent in imaging techniques such as positioning and spatial resolution. Conducting tangible measurements on dry skulls allows for precise and detailed anatomical data collection that can provide a full record of the morphology of the PH. Unfortunately, anatomical studies on dry skulls are limited by the lack of clinical context since they do not provide information about the age, gender, or medical history of the subjects. This shortcoming may limit the generalizability of the findings and their applicability to living populations.

## Conclusions

We present a comprehensive study of the morphology of the PH. To the best of our knowledge, the normal values for length, width, or angle of the PH are not yet determined. Our investigation encompasses a substantial number of pterygoid hamuli and clear measurement methods, enabling reliable and accurate results. We hope that our research will prove valuable in establishing reference standards for the dimensions of the PH and serve as a resource for future anatomical and radiological studies. As we have demonstrated, a combined anatomical, radiological, and clinical approach is essential for elucidating the implications of the pterygoid hamuli anatomy.
